# Direct Care Workforce: Where the Boys Really Are

**DOI:** 10.1093/geroni/igaa057.584

**Published:** 2020-12-16

**Authors:** Christopher Kelly, Jerome Deichert, Lyn Holley

**Affiliations:** 1 University of Nebraska Omaha, Omaha, Nebraska, United States; 2 University of Nebraska at Omaha, Omaha, Nebraska, United States

## Abstract

Purpose: This study describes the continued growth of male direct care workers (DCWs) and identifies the occupations with the greatest concentrations of male DCWs by utilizing the expanded information available in the 2018 American Community Survey (ACS). Design and Methods: Data were taken from the 1% Public Use Microdata Sample (PUMS) from the 2018 ACS. Beginning in 2018, the ACS separated the single occupation category nursing, psychiatric, and home health aides into three categories: home health aides, nursing assistants, and orderlies and psychiatric aides. Results: Between 2000 and 2018, the total number of male DCWs in the U.S. increased 118% to 474,925, with more than half (52.6%) in 2018 employed as nursing, psychiatric, and home health aides. Among these 250,139 aides, 62% (154,557) were employed as nursing assistants, 23% (57,126) worked as home health aides, and 15% (38,456) were employed as orderlies and psychiatric aides. However, 60% of all orderlies and psychiatric aides were male; this was the only occupation in the direct care workforce in which men were in the majority. Implications: The majority of male DCWs work as nursing, psychiatric, and home health aides and the new occupation classifications in the ACS reveal that while most work as nursing assistants and home health aides, the one occupation with a majority male workforce was orderlies and psychiatric aides. These findings suggest that the greatest need for male DCWs may be as orderlies and psychiatric aides, occupations in which size and physical strength are important factors.

